# Automated Neural Architecture Search for Cardiac Amyloidosis Classification from [18F]-Florbetaben PET Images

**DOI:** 10.1007/s10278-024-01275-8

**Published:** 2024-10-02

**Authors:** Filippo Bargagna, Donato Zigrino, Lisa Anita De Santi, Dario Genovesi, Michele Scipioni, Brunella Favilli, Giuseppe Vergaro, Michele Emdin, Assuero Giorgetti, Vincenzo Positano, Maria Filomena Santarelli

**Affiliations:** 1 Department of Information Engineering, University of Pisa, Via G. Caruso 16, 56122 Pisa, Italy; 2 Bioengineering Unit, Fondazione Toscana G Monasterio, Via Giuseppe Moruzzi, 56124 Pisa, Italy; 3 Nuclear Medicine Unit, Fondazione Toscana G Monasterio, Via Giuseppe Moruzzi, 56124 Pisa, Italy; 4 Athinoula A. Martinos Center for Biomedical Imaging, Massachusetts General Hospital and Harvard Medical School, Boston, MA USA; 5 Division of Cardiology and Cardiovascular Medicine, Fondazione Toscana G Monasterio, Via Giuseppe Moruzzi, 56124 Pisa, Italy; 6 Health Science Interdisciplinary Center, Scuola Universitaria Superiore ‘S. Anna”, Piazza Martiri della Libertà 33, 56127 Pisa, Italy; 7 CNR Institute of Clinical Physiology, Via Giuseppe Moruzzi, 56124 Pisa, Italy

**Keywords:** Neural architecture search, AutoML, Nuclear medicine, [18-F]-Florbetaben, Cardiac amyloidosis

## Abstract

Medical image classification using convolutional neural networks (CNNs) is promising but often requires extensive manual tuning for optimal model definition. Neural architecture search (NAS) automates this process, reducing human intervention significantly. This study applies NAS to [18F]-Florbetaben PET cardiac images for classifying cardiac amyloidosis (CA) sub-types (amyloid light chain (AL) and transthyretin amyloid (ATTR)) and controls. Following data preprocessing and augmentation, an evolutionary cell-based NAS approach with a fixed network macro-structure is employed, automatically deriving cells’ micro-structure. The algorithm is executed five times, evaluating 100 mutating architectures per run on an augmented dataset of 4048 images (originally 597), totaling 5000 architectures evaluated. The best network (NAS-Net) achieves 76.95% overall accuracy. *K*-fold analysis yields mean ± SD percentages of sensitivity, specificity, and accuracy on the test dataset: AL subjects (98.7 ± 2.9, 99.3 ± 1.1, 99.7 ± 0.7), ATTR-CA subjects (93.3 ± 7.8, 78.0 ± 2.9, 70.9 ± 3.7), and controls (35.8 ± 14.6, 77.1 ± 2.0, 96.7 ± 4.4). NAS-derived network performance rivals manually determined networks in the literature while using fewer parameters, validating its automatic approach’s efficacy.

## Introduction

Machine learning (ML) is a discipline that supports radiologists in the development of new biomarkers and better analysis of medical images towards accurate diagnosis. Among ML techniques, deep learning (DL) provides powerful methods for classification, segmentation, and recognition of medical images [1, 2]. DL is based on algorithms relying on Neural Network (NN) structures, made of several interconnected nodes, also known as neurons, that process information and automatically extract features from unstructured data [3].

NN, in general, are comprised of three main types of layers, each one composed of several nodes: the input layer, which receives data and passes it to the rest of the architecture; the hidden layers, which apply non-linear functions to the data; the output layer, that provides processing results under various formats depending on the task at hand (regression, classification).

Convolutional neural networks (CNN) are a subtype of NN, having as hidden layers three specific ones: convolutional layer, pooling layer, and fully connected layer. When using CNNs for classification tasks, convolutional and pooling layers extract features and information and feed them to the fully connected layers. These final layers, in turn, give class scores for the images. Designing and finding an appropriate neural network, a CNN in particular, can be a challenging task; in fact, most of the advances in neural network models usually require considerable hand-tuning of the neural network architecture, which is time-consuming and error-prone. Often, modifications to existing architectures are made using transfer learning, but their effectiveness is very much linked to the experience and knowledge of the researcher [4].

In recent years, auto machine learning (AutoML) has been developed to fulfill two main goals: automate the learning process from data pre-processing to model evaluation and make deep learning accessible to non-experts. An example of AutoML is neural architecture search (NAS) [5], which uses automated algorithms and techniques to find architectures that can achieve high performance while minimizing the need for manual trial-and-error. The process involves exploring a large search space of possible architectures and hyperparameters to find the most suitable configuration for the given problem.

The first NAS methods relied on reinforcement learning [6] and evolutionary learning [7] approaches, which achieved the best classification accuracy in image classification. This novel methodology has been used to accomplish some medical tasks, such as classifying skin lesions [8] or segmenting medical images for surgery planning and computer-aided diagnosis [9].

However, as far as we know, there are no studies regarding the application of this technique to the classification of nuclear medicine images, positron emission tomography (PET) in particular. This research aims to fill this gap by adopting and implementing the NAS-based evolutionary algorithm for cardiac amyloidosis (CA) classification from early acquired [18F]-Florbetaben PET images. Given a dataset that includes PET images from subjects with both light chain amyloidosis (AL) and transthyretin amyloidosis (ATTR) sub-types of CA as well as control subjects, the NAS methodology used in the present work is shown to automatically develop and evaluate the optimal network for the classification of the three data classes.

A comparison is also made with a CNN network already present in the literature, named CAclassNET [10], built with the classic methodology of manually finding an optimal network for classification through numerous hand-tuning phases of the parameters present in the network.

## Materials and Methods

### Theory

#### Neural Architecture Search

**Fig. 1:**
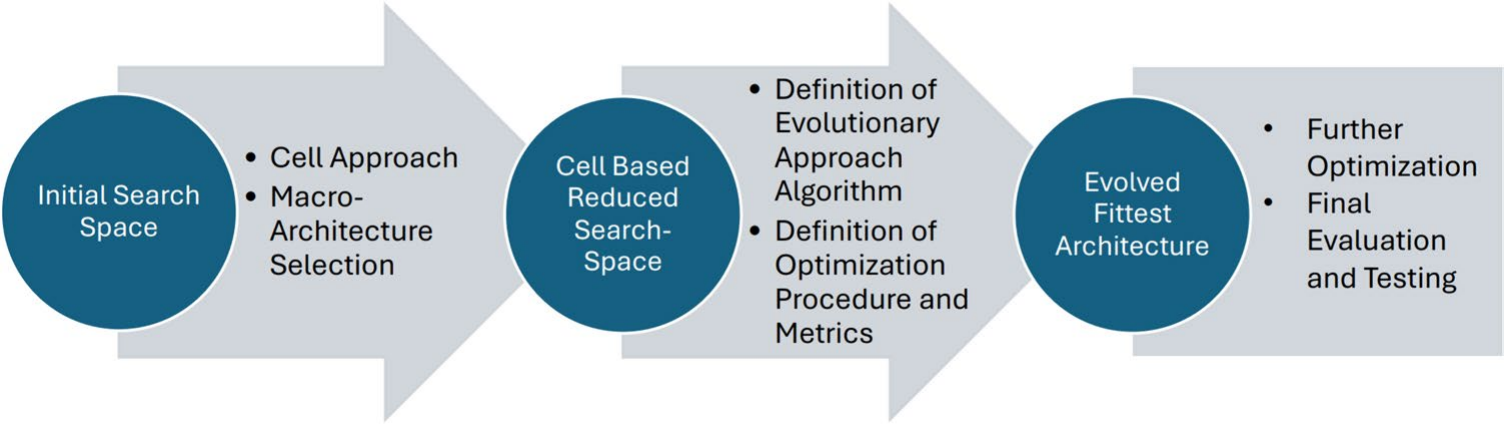
The Neural Architecture Search workflow

NAS focuses on optimizing the topology of an architecture, usually portrayed through a directed acyclic graph (DAG), where neural network operations label the nodes or edges. NAS methods are typically categorized according to three dimensions: 1. The *Search Space* **A** refers to all possible architectures that can be used for a given task; 2. The *Search Strategy*, which explores the search space by selecting a single architecture *α* (∈ A); and 3. The *Performance Estimation Strategy*, that evaluates the model’s predictive performance on unseen data and can be done, for example, using the classic training and validation approach on the data. Figure 1 gives a synthetic description of the NAS workflow followed in this work.

#### Search Space

A search space is the set of all architectures that the NAS algorithm is allowed to select. Common NAS search spaces range in size from a few thousand to over a billion architectures. Let us consider a NN as a function that, by applying operations to input variables *x*, produces output variables *y*. We can formalize it as a DAG with a set of nodes {*z*^(1)^*,z*^(2)^*,…,z*^(*k*)^*,…*} = *Z*. Let *O* be a set of operations, each node *z*^(*k*)^, except for the first one that is considered the input node, is a tensor evaluated as follows:

$${z}^{\left(k\right)}={o}^{\left(k\right)}\left({I}^{\left(k\right)}\right)$$

with *I*^(*k*)^ inputs form the sets of parent nodes and *o*^(*k*)^ (∈ *O*) operation applied to nodes. The main operations, as per [11], are convolutions, pooling, activation functions, concatenation, addition, etc. Once all the possible operations are defined, the search space can be considered either as a whole or not, giving, respectively: 1. Global search space or 2. cell-based search space. A chain and a hierarchical structure are also possible but not of interest for this work. In a global search space approach, NAS algorithms find all the components required for the entire neural network; consequently, the search space is large because the graph represents the entire network down to the single operation. Instead, in a cell-based search space approach (the one used in this work), the network is subdivided into several cells [12] with different hyperparameters (e.g., the number of filters in the first cell can be different from the number of filters in the second one). This second approach was proposed because many handcrafted architectures consist of repetitions of fixed structures called cells or blocks, which can be represented by a DAG. In this case, the network macro-architecture is manually defined [5], while the NAS approach is reserved for the micro-architecture inside each cell. Usually, two kinds of cells are stacked together repetitively: the *normal cell* that preserves the dimensions of the input; the *reduction cell* that reduces the spatial dimensions of the input.

#### Search Strategy

A search strategy is an optimization technique used to find a high-performing architecture in the search space. Once the search space has been defined, it is important to explore it using suitable approaches. There are generally two main categories of search strategies: the black box optimization–based techniques (including multi-fidelity techniques) [13, 14], and the one-shot techniques [15]. However, there are some NAS methods for which both or neither category applies. Once the search space has been defined, it is important to explore it using suitable approaches. The NAS problem can be defined as follows [11]: Let *D* be the space of all datasets, *M* the space of all deep learning models, and *A* the architecture search space, then a general deep learning algorithm Λ is defined as follows:

$$\wedge :D\;x\;A\to M$$

In this setting, an architecture *α* $\in$ *A* defines the network’s topology, parameters, hyperparameters, and regularization. Let *d* $\in$ *D* be a dataset, which is split into a training and a validation set (*d*_train_, *d*_validation_), the algorithm estimates the model *m*_*α,θ*_ $\in$ *M*_*α*_ by minimizing a loss function *L* with a regularization term *R*:

$$\wedge \left(\alpha , d\right)=\begin{array}{c}\text{arg}\;min\\ {m}_{\alpha , \theta }\in {M}_{\alpha }\end{array}\mathcal{L}\left({m}_{\alpha , \theta }, {d}_{train}\right)+R(\theta )$$

NAS has the task of finding *α*^∗^ which maximizes an objective function $f(\alpha )$ of the validation partition *d*_validation_. For example, considering the classification task, $f(\alpha )$ is usually the validation accuracy:

$${\alpha }^{*}= \begin{array}{c}\text{arg}\;max\\ \alpha \in A\end{array} f(\alpha )$$

Here, the function *f* is considered only dependent on $\alpha$ as all the other settings are considered fixed during the NAS procedure. Several approaches exist in literature to explore the search space, such as random search, reinforcement learning [6, 16], gradient-based optimization differentiable ARchiTecture search (DARTS) [17], and evolutionary algorithms [7]. Evolutionary algorithms use the essential components of a genetic optimizer to find the best neural network [7, 18, 19]. The approach described in [19] and used in the present work requires the definition of a set of primary operations and mutation rules; the overall macro-architecture is also predetermined. Each architecture consists of a sequence of normal cells (in a stack of *N* cells) and reduction cells. For each stack of normal cells, the number of convolutional filters is equal to *F*; this number is then doubled after each reduction cell. The goal of this algorithm is to find the best reduction and normal cells (micro-architecture). Then, the search strategy works as follows: after an initial selection of *P* architectures, each consisting of a repetition of normal and reduction cells, the validation accuracy is evaluated by training each model from scratch. After, the evolution algorithm is applied. With *C* as the number of generations (number of steps of the evolutionary algorithm), a sample of *S* models is randomly selected with replacement. The model with the highest accuracy among the *S* selected samples is then picked as the parent and mutated. The following three mutation rules are chosen according to [19]:

1. *Operation mutation*: once a cell and a pair of hidden states are selected, one of the two operations is changed (probability: 0.475).
2. *Hidden state mutation*: once a cell and a pair of hidden states are selected, one of the two hidden states is changed (probability: 0.475).
3. *Identity mutation* (in which nothing changes) is also possible but with a lower probability (0.05).

**Fig. 2:**
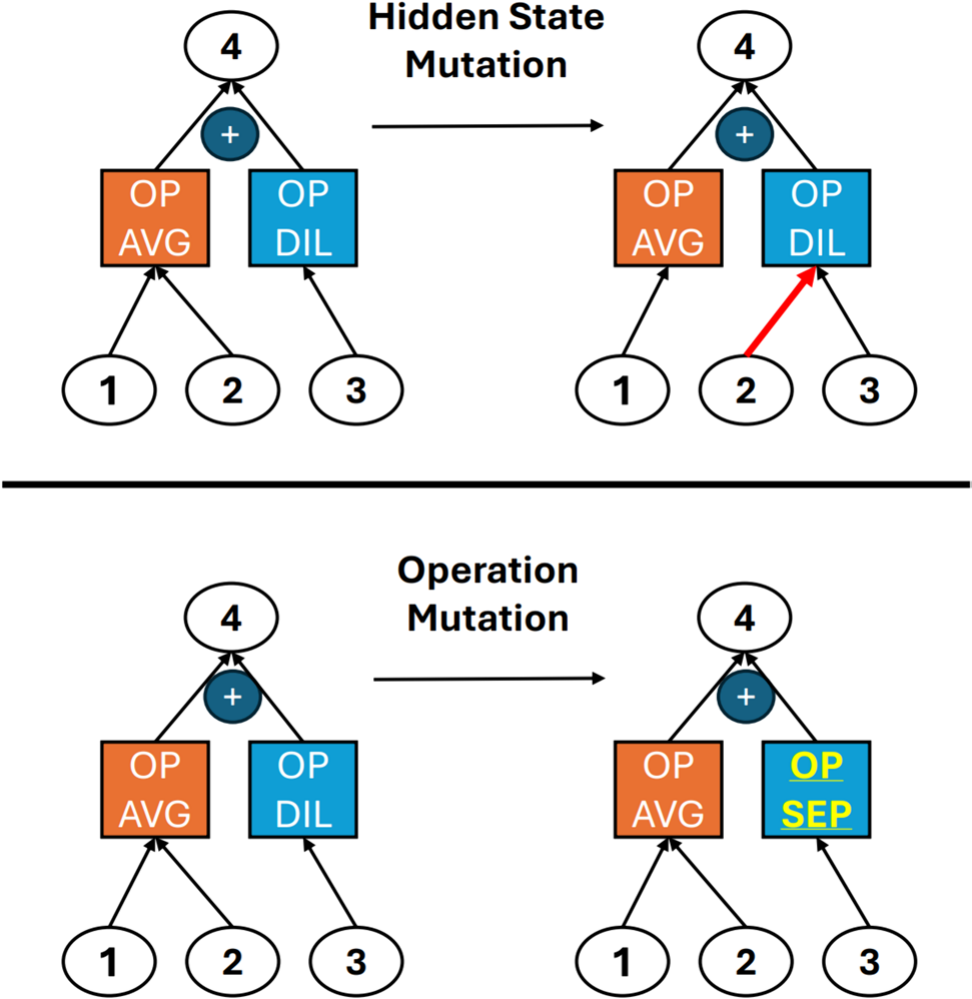
Visual representation of hidden state and operation mutations inside a cell. Hidden state mutation (top): hidden state 2 connection to operations is changed; Operation mutation (bottom): the convolutional dilatation operation (OP DIL) is changed into a convolutional separable operation (OP SEP), the average pooling (OP AVG) is left unchanged

At each step, a mutation is randomly selected and then applied to a specific cell (normal or reduction) (Fig. 2). The offspring is then trained, and its validation accuracy is evaluated. The oldest model is then removed from the population to keep the size *P* constant.

To speed up the search, the different architectures are trained for a smaller number of epochs. Then, only a subset, consisting of the best models, is selected, eventually augmented (by increasing *N* and/or *F*), and trained for a higher number of epochs.

#### Performance Estimation Strategy

A performance estimation strategy is any method used to quickly predict the performance of neural architectures to avoid fully training the architecture. For example, while we can run a discrete search strategy by fully training and evaluating architectures chosen throughout the search, using a performance estimation strategy such as learning curve extrapolation can greatly increase the speed of the search. During the search process, it is necessary to evaluate the performance of the candidate architecture. The easiest approach that can be used is training a neural network from scratch and evaluating its performance on the validation set. Since this approach is computationally heavy and requires a lot of GPU time, different approaches are proposed in the literature to speed up the performance estimation [5]. One of the most used methods that we used in the present work is the *lower fidelity estimates*, consisting of estimating the performance of the network from the learning curve trained for fewer epochs and from the relevant hyperparameters [20, 21].

### Image Data

#### Cardiac Amyloidosis Diagnosis

CA is a cardiomyopathy associated with the deposition of protein fibrils in the extracellular space of the heart [22]. Several types of amyloidosis can usually be distinguished. The most relevant in cardiac amyloidosis are immunoglobulin light-chain amyloidosis (AL) and transthyretin-related amyloidosis (ATTR). The main problem of this disease is that the early clinical symptoms can be confused with other conditions such as hypertensive heart disease or heart hypertrophy secondary to aortic valve stenosis. Moreover, these two subtypes of amyloidosis require different therapies: AL patients are usually treated with chemotherapy or stem cell transplantation, while ATTR patients are subjected to small RNA-silencing molecules or stabilizers [23, 24]. Therefore, it is very important not only to diagnose the presence of amyloidosis as soon as possible but also to be able to characterize which subtype it is. Nowadays, the diagnosis of ATTR in the absence of a monoclonal disease can be obtained by scintigraphy with bone-seeking agent labelled with 99mTc. Instead, when a monoclonal component in serum and/or urine is present or for the diagnosis of AL, a histologic approach, often by endocardiac biopsy is required [25, 26]. The major drawback of cardiac biopsy is the risk associated with the invasiveness of the technique. Therefore, researchers are trying to use non-invasive methods such as medical imaging to obtain the information needed for early diagnosis [26, 27]. In PET imaging, characterization of the CA can be performed by the evaluation of specific quantitative indexes such as standardized uptake value (SUV) SUV_max_, SUV_mean_ and molecular volume obtained with [18F]-Florbetaben by acquiring early and late static 3D images of the thorax after the injection of the radiopharmaceutical [28–30]. Alternatively, a dynamic approach can also be taken to evaluate indexes that allow CA diagnosis [31]. Being able to make an accurate differential diagnosis from a single static PET images acquired in an early phase, i.e., after a few minutes from the injection of the tracer, should have the double advantage of reducing the waiting time for the examination to be performed (for the patient) and obtaining a better organization for the nuclear medicine laboratory. Accordingly, in the present work, a set of cardiac amyloidosis images, consisting of 3D static PET acquired 15 min after the injection of the [18F]-Florbetaben, was used to test the goodness of the proposed approach.

#### Subjects and Cardiac PET Data Acquisition

A total of 47 subjects are included in this retrospective study, including 28 patients with systemic amyloidosis and heart involvement (13 patients with AL and 15 patients with ATTR cardiac amyloidosis, respectively) and 19 control patients with the clinical suspicion of CA, that received an alternative diagnosis, such as left-ventricle hypertrophy secondary to aortic-valve stenosis, primary hypertrophic cardiomyopathy, or hypertensive cardiac hypertrophy. Patients with ischemic heart disease, chronic liver disease, or severe renal failure were not included in the study. Diagnosis of CA was based on clinical examination, biomarkers positivity, electrocardiogram, echocardiography, bone-scintigraphy, cardiac magnetic resonance (CMR), and histological evidence of amyloid deposition according to the most recent cardiological evidence and guidelines [32, 33]. Further details on patients’ characteristics are described in [10]. The study was approved by the institutional ethics committee and the AIFA (Agenzia Italiana del Farmaco) committee; all subjects signed an informed consent form. The study complied with the Declaration of Helsinki. Each subject underwent PET/CT examination. A Discovery RX VCT 64-slice tomography (GE Healthcare, Milwaukee, WI, USA) was used for image acquisition. Firstly, a low-dose-computed tomography (CT) (tube current 30 mA, tube voltage 120 kV, effective dose of 1 mSv), covering the heart, was performed for attenuation correction. Then, 40 min of PET data were acquired, starting at the time of injection of an intravenous bolus of [18F]-Florbetaben (300 Mbq/1 ml) followed by a saline flush of 10 ml (1 ml/s). The raw PET list mode data file was histogrammed between 15 and 20 min of post-injection, to create a single static sinogram. Then, 3D static PET images were reconstructed using the ordered subset expectation maximization (OSEM) iterative algorithm with three iterations and 21 subsets. Each 3D volume consisted of 47 axial slices with a 128 × 128 pixels matrix.

### Image Pre-processing

**Fig. 3:**
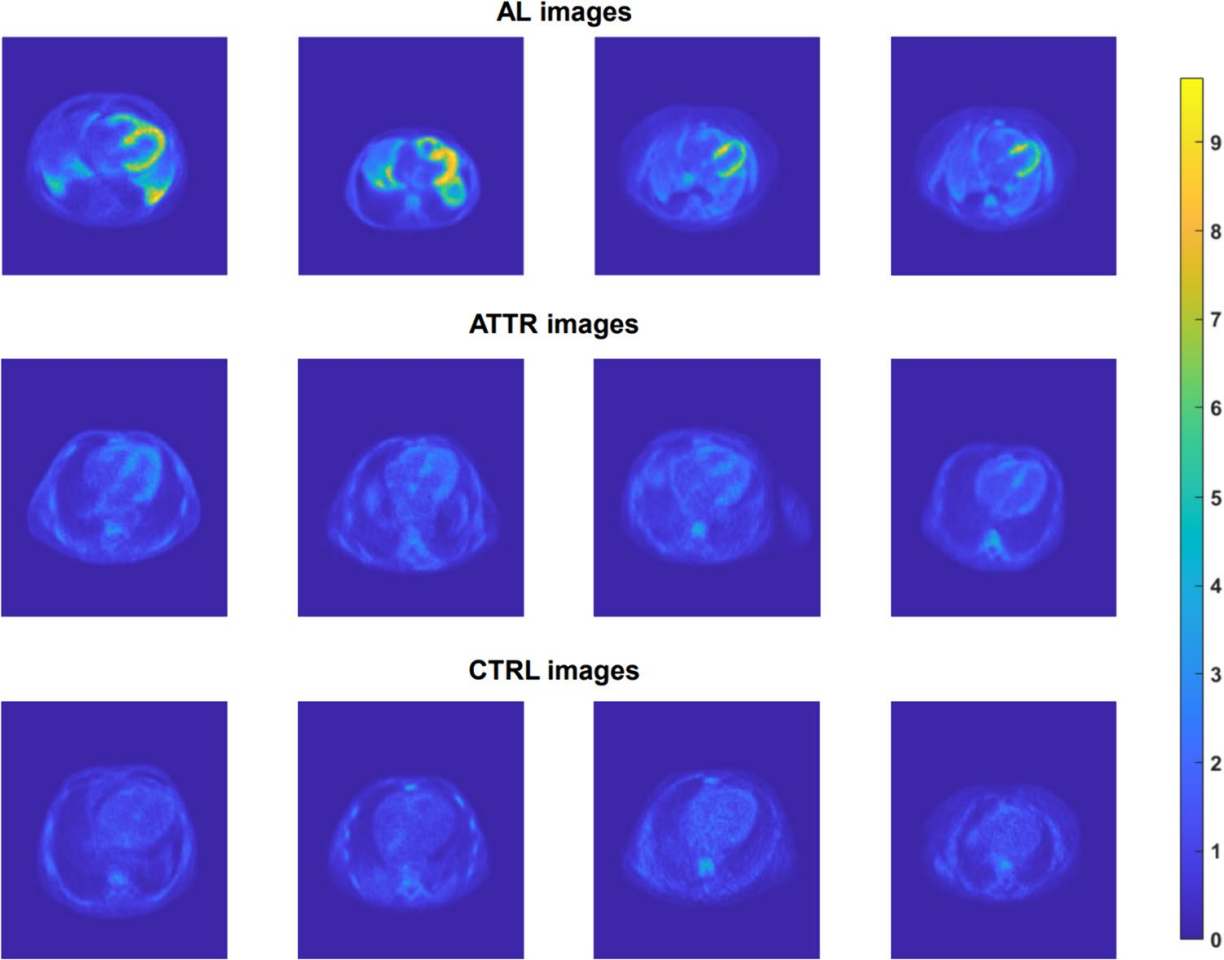
Example of images from the different classes: values are in SUV

From the reconstructed axial slices of each volume, only those covering the heart were taken into consideration in the study; accordingly, for each patient, the number of images considered varied from a minimum of eight to a maximum of 19 slices. In addition, image cropping was performed. The final dimensions of the images are of 77 × 104 pixels. A total of 592 2D images (193 from controls, 240 from AL-subtype patients, and 159 from ATTR-subtype) have been considered in the study. In Fig. 3, examples of reconstructed and cropped images from AL, ATTR, and CTRL subjects are shown. To achieve better performance during training and avoid overfitting, data augmentation has been implemented. Following, an affine transformation was used [10], being recognized in literature as the most suitable methodology for the augmentation of data sets in medical imaging [34]. Specifically, each image was randomly translated in both row and column directions of a maximum of 10 pixels and randomly rotated of maximum ± 10°. The affine transformation was applied ten times for each input image. The data augmentation is performed as a one-time preprocessing step and only on the training set. To avoid data leakage when evaluating the results, data splitting was performed at the patient’s level, avoiding the presence of slices from the same subjects both in the training/validation and the test set. After data augmentation, the overall dimensions of the sets are the following:

- The training set consists of 384 images augmented to 3840 (10 × data augmentation; 1550 AL, 1010 ATTR, 1280 CTRL).
- The validation set consists of 96 images (40 AL; 30 ATTR; 26 CTRL).
- The test set consists of 112 images (45 AL; 33 ATTR; 34 CTRL).

### Hardware and Software Specs

The overall algorithm is run on a PC, with Ubuntu Operative System 22.04.3 LTS, equipped with a Core i7 4790k 4-core CPU, 32GB of Ram and an Nvidia Titan Xp GPU with 12 GB of VRAM. The algorithm is implemented in Python 3.9.13 using the Anaconda environment 22.9.0 with the respective libraries. Pytorch 1.13.1 with CUDA 11.7 and CuDNN 8.5 was used for the core DL development.

### Implementation of the Algorithm and Methods Detail

The approach used to classify the datasets is based on the method described in “Theory”.

#### Choice of the Primitive Operations

The primitive operations that can be used to build a normal or a reduction cell have been selected based on [9] and [19]. To avoid redundancy, convolutions, max pooling, and mean pooling were restricted to 3 × 3; indeed, [9] shows that larger kernel sizes like 5 × 5 and 7 × 7 can be substituted by stacking appropriate 3 × 3 convolutions. In this way, each operation possesses distinct properties that cannot be substituted by others. The chosen operations are defined through a dictionary. Following [12], 1 × 1 convolutions are inserted to ensure equal dimensions of the two hidden input states. Each convolution consists of a sequence of Conv-ReLU-batch normalization. Batch normalization is a popular technique used in neural networks to improve performance and stability. This is achieved by normalizing the output of a layer to have a mean of zero and a standard deviation of one [35]. This allows the network to learn more efficiently and prevents overfitting.

#### Implementation of the Evolutionary Algorithm

To determine the best model for the provided datasets, some parameters were set.

**Fig. 4:**
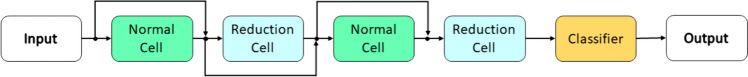
Structure of the architecture we are looking for

- The number of filters of the first cell (*F*) (this number is doubled before each reduction cell) was initially set equal to 4.
- The number of operations for each cell. For example, *n* operations correspond to *n-1* hidden states: 2 as inputs, one as output, and the remaining *n-4* are generated by applying the selected operations to the previously selected hidden states. The number of operations was set to 6.
- The number of classes for the classification task: equal to 3 corresponding to CTRL class (i.e., control subjects), AL and ATTR classes.
- The number of input channels is equal to one since the PET images are grayscale.
- The number of layers of the architecture is 4, as shown in Fig. 4.
- The number of starting architectures **P** is 100, with 900 evolutionary steps **C** (in each step 1 sample was mutated (**S**)).

An example for the first convolutional operation in the normal cell could be:

**x** = Conv2D(**input_tensor,** *F* = 4, kernel_size = (3, 3), strides = (1, 1), padding = ‘same’)

**x** = ReLU(**x**)

**x** = BatchNormalization(**x**)

**Fig. 5:**
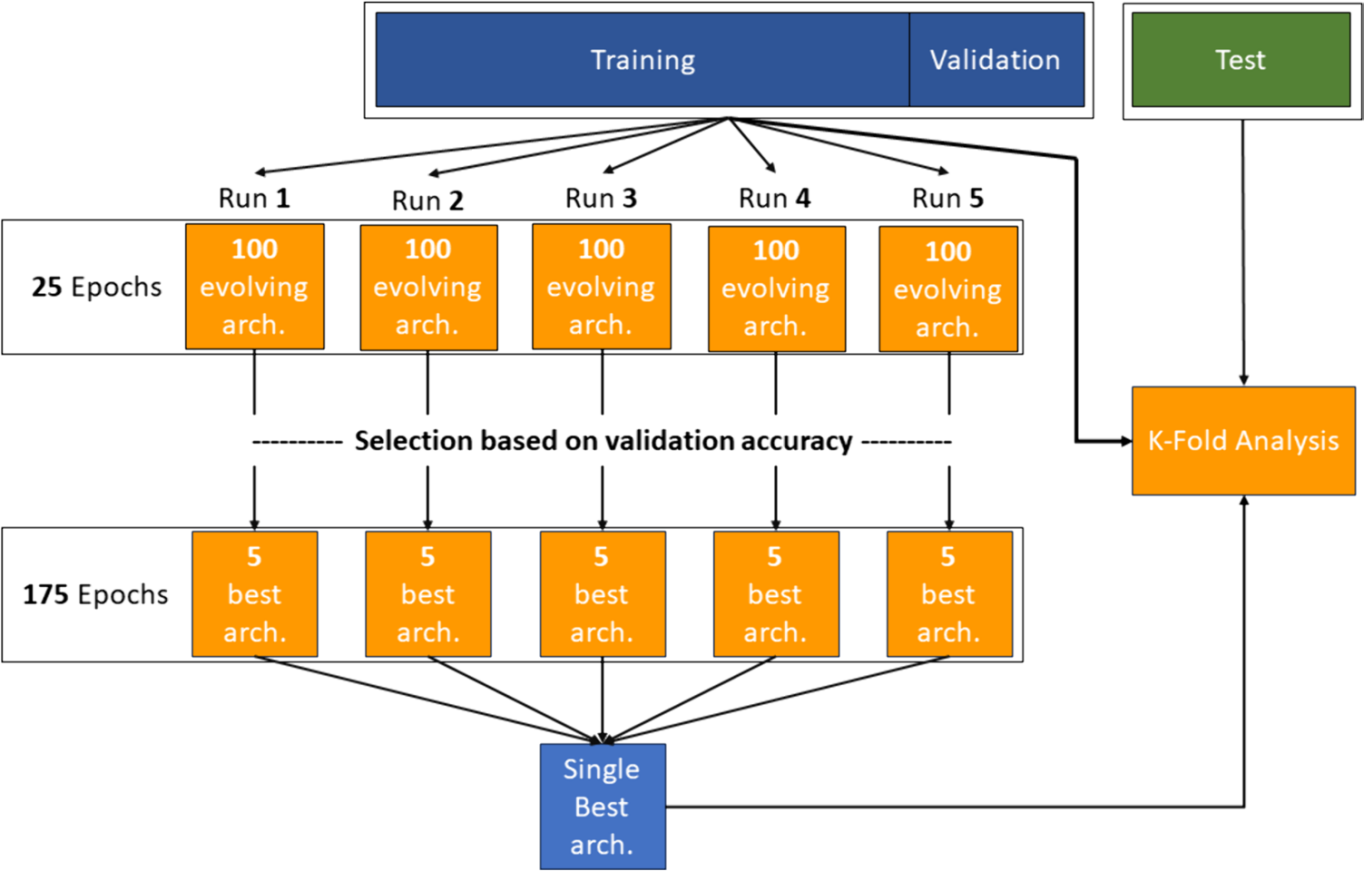
Overall performance estimation strategy and model selection

The NAS algorithm was run five times (Fig. 5). For each run, a first training step using 25 epochs was performed on a population of 100 evolving individuals, maximizing the overall classification accuracy. In the second step, the best five architectures underwent a further 175 epochs training. Hence, 5 × (P + C) = 5000 individuals were generated in the first step and 25 (5 × 5) were more deeply analyzed in the second step. In the final step, the best individual (i.e., the one with the higher overall accuracy) was identified. Once the best model is selected, a stochastic *k*-fold validation of the best model is performed using five random splits of the training/validation dataset. All the training was done using the Adam optimizer, with a learning rate of 1e-3, cross-entropy loss, and a batch size of 32. Detailed values of the hyperparameters used for the architecture search algorithm are shown in Table 1.

**Table 1:** Hyperparameters for the architecture search algorithm

Hyperparameter	Value
Starting number of filters **F**	4
Per cell operations	6
Number of classes	3
Number of channels	1
Number of layers	4
Number of starting architectures **P**	100
Number of evolutionary steps **C**	900
Number of mutated samples per step **S**	1
Number of training epochs	25
Number of further training epochs	175
Batch size	32
Loss function	Cross-entropy loss
Learning rate	1e-3
Optimizer	Adam (default parameters)

Pseudocode for the implemented algorithm is provided below. From top to bottom: initialization, initial model population creation, evolution process, final training of the best architectures and output.
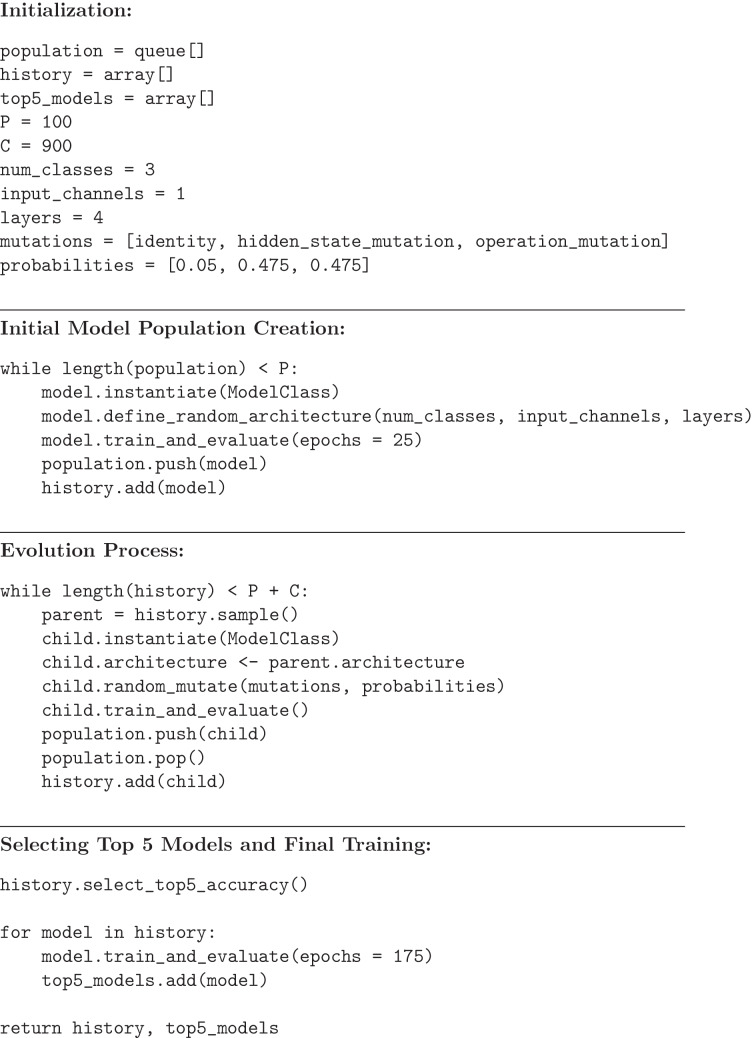


### CAclassNET as Handcrafted Neural Network for Comparison

To evaluate the goodness of the net obtained by the NAS methodology, a comparison was made with the CNN, named CAclassNET, previously proposed by the authors in [10]. In the present work, the CAclassNET was newly implemented by using Python and Pytorch facilities (in [10], it was implemented in Matlab), for a better comparison between the two networks, and trained with the optimized hyperparameters described in [10]. The training was then repeated five times to statistically evaluate the performance of the classifier on the provided dataset.

## Results

The initial and final validation accuracy results for each individual architecture among the best five are reported in Table 2 for each run.

**Table 2:** Best five individuals for each of the five runs

Individual #		Initial validation accuracy (25 epochs)	Final validation accuracy (175 further epochs)
1st RUN	5	75.00%	72.92%
	250	65.63%	*84.38%*
	421	63.54%	87.50%
	688	*63.54%*	67.71%
	990	78.13%	75.00%
2nd RUN	**363**	76.04%	***90.63%***
	376	68.75%	79.16%
	849	80.21%	84.38%
	878	85.42%	82.29%
	887	81.25%	77.08%
3rd RUN	372	85.42%	82.29%
	487	66.66%	*89.58%*
	562	76.04%	85.41%
	666	65.63%	80.21%
	995	82.29%	83.33%
4th RUN	49	69.79%	69.54%
	129	71.88%	77.08%
	268	72.91%	78.13%
	342	65.63%	80.21%
	929	73.96%	*85.42%*
5th RUN	8	59.38%	*80.21%*
	472	73.96%	67.71%
	602	70.83%	76.04%
	669	77.08%	79.17%
	799	77.08%	65.63%

**Fig. 6:**
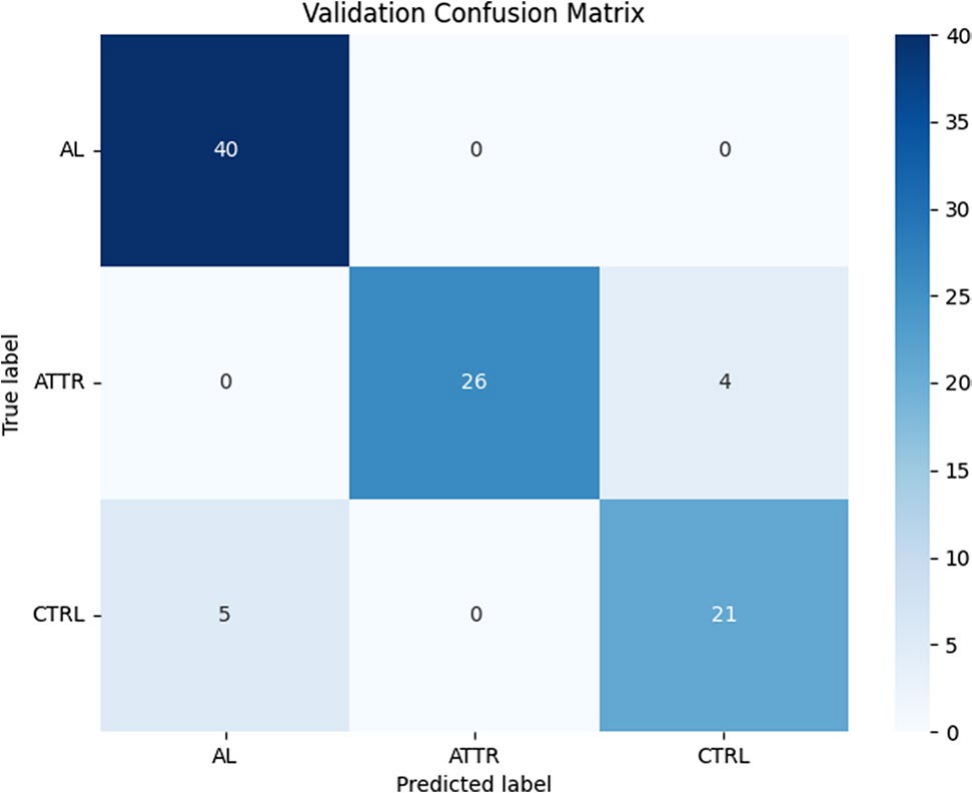
Confusion matrix on the validation set for the best model

Accuracy values were normally distributed (*p* = 0.485, Shapiro-Wilkinson test). One-way analysis of variance (ANOVA) detected no significant accuracy difference (*p* = 0.829) in the five final validation runs (Table 2). Tukey test has been used to detect anomalous observations in accuracy values, and no outliers have been detected. Of the five runs, the second run yielded the best validation accuracy, achieved by individual 363, with a final accuracy of 90.63%. The relevant confusion matrix for the validation set is shown in Fig. 6. According to such results, further deep analysis was performed on this net (NAS-Net in the following).

**Fig. 7:**
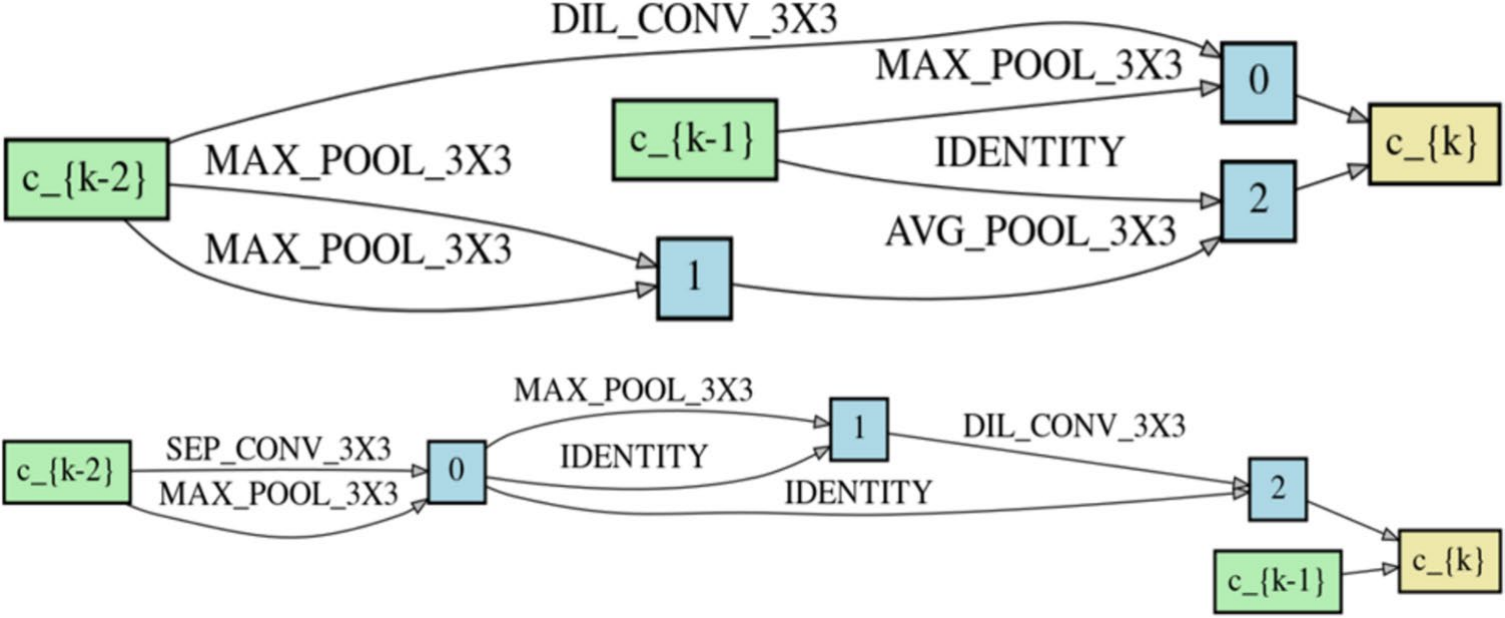
Graphs describing the architecture of the normal (top) and reduction (bottom) cell

The structure of the normal and reduction cells is shown in Fig. 7; the first two hidden states, *c*_*k*−2_ and *c*_*k*−1_, represent the two inputs of each cell, while *c*_*k*_ represents the output state.

As shown in Fig. 7, two kinds of convolution are used: dilated convolutions (DIL_CONV) and dilated separable convolutions (SEP_CONV). Each convolution operation consists of a sequence: 1. convolution; 2. ReLU; 3. batch normalization (BN). For separable convolutions, these operations are repeated twice [12]. The NAS-Net was trained five times, splitting the training and validation entries differently in a stochastic manner to statistically evaluate the performance of the classifier. For each run, the parameters were reset.

**Fig. 8:**
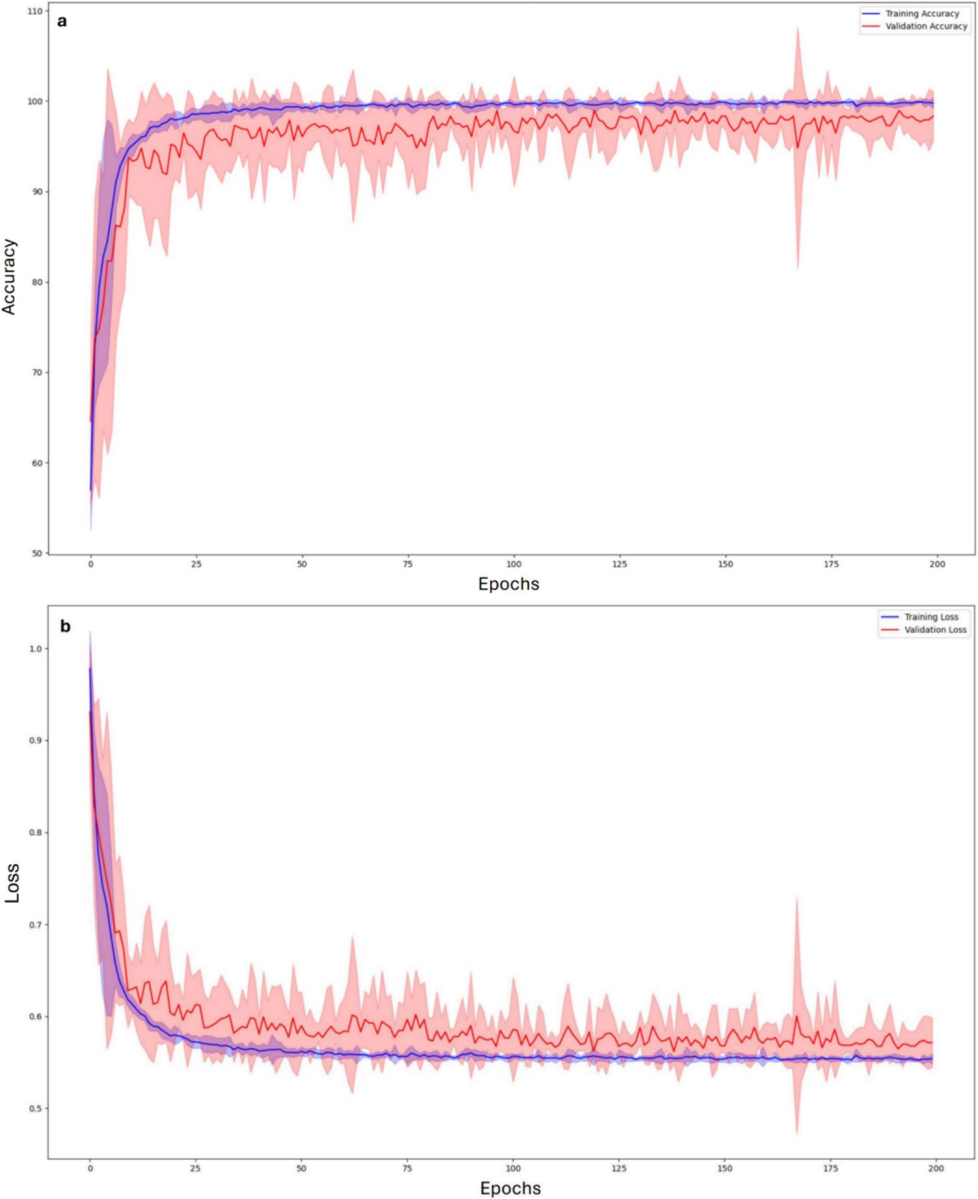
Average training (blue) and validation (red) accuracy (**a**) and loss (**b**) with 95% confidence intervals (shaded areas). On the *x*-axis the epochs

Figure 8 shows the validation and training loss of the classifier over epochs; continuous lines are the mean values of the five runs, and shadowed regions cover 95% of the confidence interval. For each run, the performance of the NAS-Net on the test set (unseen data) was also evaluated.

**Fig. 9:**
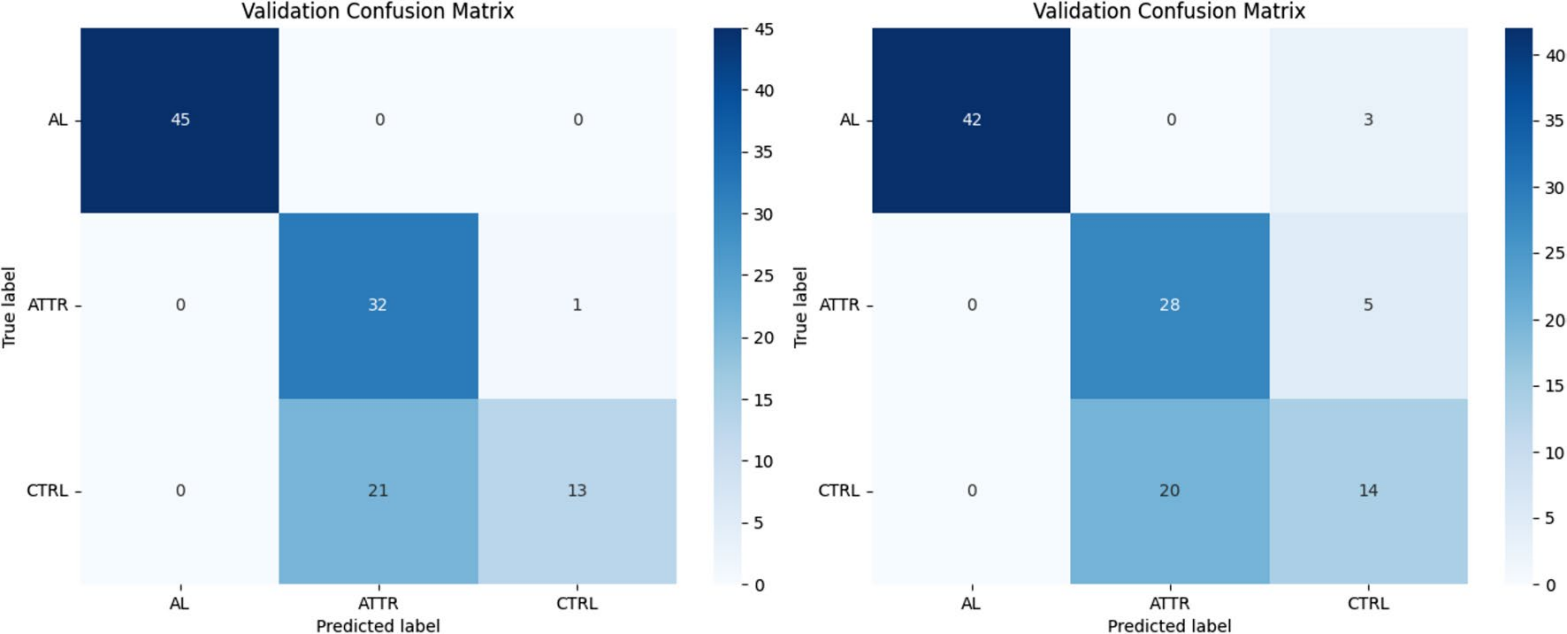
Best (left) and worst (right) confusion matrices obtained during two of the five runs

Figure 9 shows two examples of confusion matrices obtained during the different runs (the best and the worst runs, respectively). Table 3 summarizes the overall classifier performances, evaluated in terms of sensitivity, specificity, and accuracy. From repeated measurements ANOVA analysis, it results that sensitivity and specificity values in all three comparisons (i.e., AL vs. ATTR, AL vs. CTRL, and ATTR vs. CTRL) as well as accuracy values in AL vs. ATTR and AL vs. CTRL, are significantly different (*p* < 0.001); no significant difference was detected between ATTR vs. CTRL accuracy values (*p* = 0.173). The overall mean accuracy of the best classifier for the test set was 76*.*95% (± 2*.*13%). The time needed for a single run of the evolutionary algorithm and to evaluate the 5 best architectures was, on average, about 12 h and 30 min. Every subsequent retraining of the best model required about 20 min.

**Table 3:** Performance of the NAS-Net model (%)

Class	Sensitivity	Accuracy	Specificity
AL	98.7 ± 2.9	99.3 ± 1.1	99.7 ± 0*.*7
ATTR	93.3 ± 7.8	78.0 ± 2.9	70.9 ± 3.7
CTRL	35.8 ± 14.6	77.1 ± 2.0	96.7 ± 4.4

### Comparison with the Handcrafted Neural Network

The average accuracy of the CAclassNET was 99.38% for the training set and 87.35% for the validation set. Table 4 shows the performance of the handcrafted classifier as measured by sensitivity, accuracy, and specificity. Similarly to the results of Table 3, also for Table 4, the ANOVA analysis was performed: sensitivity and specificity values in all three comparisons, as well as for accuracy values in AL vs. ATTR and AL vs. CTRL, are significantly different (*p* < 0.001); no significant difference was detected between ATTR vs. CTRL accuracy values (*p* = 0.8). The overall accuracy on the test set was 79*.*21% ± 3*.*4%. The performances in terms of sensitivity, accuracy, and specificity are better than those of a doctor with more than 10 years of experience in cardiac nuclear medicine in fact, they resulted to be as follows [10]: sensitivity, specificity, and accuracy equal to 0.533, 0.744, and 0.673 respectively for AL patients, 0.314, 0.802, and 0.665 for ATTR patients, 0.562, 0.667, and 0.627 for CTRL.

**Table 4:** Performance of the CAclassNET classifier (%)

Class	Sensitivity	Accuracy	Specificity
AL	99.0 ± 1.6	99.6 ± 0*.*6	100.0 ± 0*.*0
ATTR	76.2 ± 14.0	79.6 ± 3.5	80.1 ± 5.6
CTRL	55.8 ± 11.0	79.2 ± 3.3	89.4 ± 6.2

Table 5 summarizes the differences between the two models in four aspects: number of parameters, time to define an architecture, training time, and classification time of a new image. Regarding accuracy at the subject level, both the architecture developed using the NAS method and CAclassNET are able to consistently and correctly identify 8 (3 CTRLs, 3 ALs, 2 ATTRs) out of the 11 (5 CTRLs, 3 ALs, 3 ATTRs) subjects in the test dataset. Note that ALs are always correctly classified.

**Table 5:** Comparison between the best architecture discovered by the NAS algorithm (NAS-Net) and CAclassNET

Features	NAS-Net	CAclassNet
Number of parameters	2.763 × 10^3^	93.827 × 10^3^
Implementation time	~ 8 h per 1000 architectures evaluated	days/weeks
Training time (200 epochs) [s]	224.67 (≃ 3*′45″*)	187*.*06(≃ 3′7″)
Classification time of a new image [ms]	6*.*4	3*.*4

## Discussion

### Contribution of This Work

The main objective of this study was to demonstrate the effectiveness of the neural architecture search algorithms for medical image classification, early acquired [18F]-Florbetaben PET images in particular. The use of NAS methods for defining the best model for image analysis has the great advantage of greatly reducing the operator’s contribution in defining the structure and parameters to be used, making these operations almost completely automatic. Therefore, the effort required to design the deep learning models is reduced, and researchers can focus on other aspects, such as data pre-processing and model tuning, improving the performance of the models found. Unlike ordinary images, in which large databases are available online, the analysis of medical images using deep learning methods is often challenging due to privacy concerns and the rarity of certain pathologies. This is especially true for PET images, where datasets are increasingly limited. In literature, some attempts have been made, and some methods based on the NAS approach have been proposed on medical images, mainly for image segmentation [9], but, as far as we know, there are no studies on the classification of cardiac amyloidosis from early acquired [18F]-Florbetaben PET images; in fact, we can state that only the authors have implemented a CNN that performs this task [10], but not using NAS technology.

### Methodology

The cell-based search space method was selected in this work. This search space consists of architectures composed of repeating blocks of two main types: normal and reduction cells. Each cell consists of a DAG that describes how the different states are combined to form a new state using primary operations. Search space is then explored using an aged evolutionary algorithm: the oldest individual in history dies at each generation. The results obtained after running the proposed NAS approach five times, after 25 epochs had an accuracy from a minimum of 59.38% (see Table 2, fifth run) to a maximum of 85.42% (see Table 2, second and third run), with a mean value of 73.04%. Therefore, already after 25 epochs, the NAS approach has given quite promising results. But the results obviously improved after a further 175 epochs, bringing the accuracy to a minimum value of 65.63% (see Table 2, fifth run) and a maximum of 90.63% (Table 2, second run) with a mean of 79.24%. The model giving the highest accuracy has been considered as the network for cardiac amyloidosis classification. The proposed two-step approach was designed to obtain a reasonable processing time for individual selection. The structure of the best network model obtained by the proposed NAS approach (NAS-Net) is shown in Figs. 4 and 7; the behavior of the architecture as a graph is evident both for the structure as a whole and for the individual cells. The identity operations in the reduction cell (Fig. 7) are introduced to maintain the network’s depth constant.

### Results

The confusion matrix obtained for the network with higher validation accuracy (see Fig. 6) demonstrates that the determination of the cardiac amyloidosis AL class is optimal, with some uncertainty between the ATTR class and controls. Training and validation accuracy trends of the selected model, shown in Fig. 8, have a typical shape in network analysis: both curves increase over epochs as the model learns to make more appropriate predictions on both sets. A gap exists between training and validation curves being training higher than validation; this is expected and mainly due to the low number of data available as it happens to all imaging techniques that require, for example, the use of ionizing tracers and/or invasive maneuvers for which images are acquired only if strictly necessary. However, it is worth to note that at 200 epochs the accuracy for validation data is anyway quite high, having the mean value equal to 98.3% (see Fig. 8). Also, for training and validation losses both curves decrease over epochs. This is an indication that the model is learning to make more accurate predictions for the training and validation set. Both confusion matrices (Fig. 9) and sensitivity, accuracy, and specificity values (Table 3) show that the network well determines AL cardiac amyloidosis patients. In contrast, ATTR amyloidosis patients and controls are sometimes incorrectly diagnosed, with NAS-Net privileging sensitivity for ATTRs (93.3%) and specificity for CTRLs (96.7%). This is well documented in literature where it is asserted that the cardiac PET imaging using [18F]-Florbetaben well characterizes the presence of type AL amyloidosis, while it is not able to determine the ATTR and to distinguish it from other pathologies or from the non-presence of cardiac pathology [30]. This is even true when considering early acquired images, i.e., at 15 min after injection [10], as it is in our study. On the other hand, by reducing the classification task to AL vs. non-AL subjects, the performance of the discovered classifier is optimal, well identifying subjects affected by CA of type AL. To demonstrate the validity of the proposed approach, that is, it automatically generates an optimal network that is comparable with the best one obtainable manually, a comparison has been made with a state-of-art handcrafted CNN, carefully tuned on the same data set. In fact, from Tables 3 and 4, we can see that the two networks showed a similar performance pattern, with very good sensitivity, accuracy, and specificity values for the AL class and lower values for ATTR and CTRL classes. All values were >70% except for the CTRL sensitivity value for both networks. Moreover, in Table 5, the performances of the two nets are compared, showing a 40 times higher value of the number of parameters for the CAclassNet, while the training processing time and the classification time of a new image are slightly higher for NAS-based net. Overall, we can say that the NAS-based algorithm found a model whose performance is comparable to that available in the literature. Indeed, it correctly discriminates between AL and non-AL images but shows intermediate performance in classifying ATTR and CTRL.

### Advantages, Disadvantages, and Limitations

The implementation of this approach made it possible to clearly highlight both the advantages and disadvantages of this technique. A great advantage is that the best architecture can be automatically identified that is better suited to the specific problem at hand. The disadvantage is the computational cost since multiple neural networks must be trained and evaluated to find the best one. In this work, to reduce this weakness, we reduced the number of training epochs to speed up the process of exploring search space. Then, the best architectures were trained for more epochs to find the best-discovered model. However, it is worth noting that such optimal parameters search phase, which requires high processing times, in conventional methods has still to be performed, and it is done with the continuous contribution of the operator and, therefore, not automatically. While the definition and training of CAclassNET required repeated architecture evaluations and, only subsequently, hyperparameter tuning, the evolutionary algorithm set for the NAS network automatically selects the best architecture once the hyperparameters are specified (Table 1). In the present work, these hyperparameters were set according to empirical knowledge in NAS literature, reducing the time required for hyperparameter search. One hidden cost that could also be considered is the human-production cost associated with the implementation of the code used for this work, which, however, can be reused as an asset for future model development (code once, run forever).

The network generated here with the NAS method is aimed at classifying amyloidosis from PET data; in the present work, we have not evaluated whether this net can be adapted to other image classification tasks. Anyway, we suppose that, either by re-running the evolutionary algorithm on new data/with different hyperparameters or with appropriate network modifications typical of transfer learning, the methods shown in this work could be used for the development of any convolutional model for the classification of biomedical images (or even other tasks, with appropriate modifications).

One limitation in this work is the low amount of data: data relevant to 47 subjects are considered, for a total of 592 2D PET images. This is not a lot of data for deep learning analysis, as it is often the case for biomedical images. But one of the purposes of this work was precisely to evaluate whether the NAS methodology was efficient even when the data available is rather limited.

## Conclusions

In the present work, the NAS approach was applied to classify medical images. In particular, the main objective has been to evaluate the possibility of automatically finding an optimal network for the classification of cardiac amyloidosis from [18F]-Florbetaben PET images acquired 15 min after injection. The results obtained are very promising, being very similar to those available in the literature for CNNs designed manually, while for the proposed approach this task was carried out completely automatically.

## Data Availability

Data used in this article are not available due to it being property of the healthcare institution.

## Code Availability

Developed code is available upon request to the corresponding author.
